# A quantitative immunoassay for lung cancer biomarker CIZ1b in patient plasma

**DOI:** 10.1016/j.clinbiochem.2016.11.015

**Published:** 2017-04

**Authors:** Dawn Coverley, Gillian Higgins, Daniel West, Oliver T. Jackson, Adam Dowle, Aidan Haslam, Eve Ainscough, Rebecca Chalkley, John White

**Affiliations:** a Cizzle Biotech, University of York, YO10 5DD, UK; b Department of Biology, University of York, YO10 5YW, UK; c Department of Respiratory Medicine, York Teaching Hospital NHS Foundation Trust, YO31 8HE, UK; d Hull-York Medical School, University of Hull, HU6 7RX, UK

**Keywords:** Lung cancer, CIZ1b, Blood test, Immunoassay, Biomarker

## Abstract

**Objectives:**

Non-invasive tests for early detection of lung cancer are an important unmet clinical need. CIZ1b plasma biomarker can discriminate stage 1 lung cancer from within high-risk groups with clinically useful accuracy, with ROC AUCs in excess of 0.9 for two independent retrospective cohorts, and could therefore meet this need. Our aim was to characterise the native state of the biomarker and develop a quantitative immunoassay.

**Design and methods:**

Selective denaturation, preparative electrophoresis and mass spectrometry of human plasma were used to characterise the biomarker and interaction partners. A sandwich ELISA was generated, and specificity for CIZ1b biomarker tested on lung cancer patient plasma.

**Results:**

CIZ1b biomarker is a denaturation-resistant complex between a C-terminal fragment of CIZ1 bearing the CIZ1b epitope specified by alternative splicing of exon14, and fibrinogen alpha chain. Reconstitution of the biomarker epitope with purified fibrinogen and CIZ1b, but not CIZ1a (non-alternatively spliced exon 14) confirmed the specificity of the results. The endogenous complex is highly stable in lung cancer plasma and can be quantified by pairing of a CIZ1b exon-junction specific antibody with detection of fibrinogen. Application of this sandwich ELISA to a prospectively collected development set of plasmas reveals the same level of accuracy as the western blot used to validate the discriminatory capability of the biomarker.

**Conclusions:**

Unexpected and unusual molecular structure of CIZ1b in native plasma has complicated immunoassay design, and delayed translation of this promising biomarker. However, CIZ1b can now be measured using a high-throughput, hospital-friendly sandwich ELISA format, overcoming an important barrier to further clinical development and application of this blood test for early stage lung cancer.

## Highlights

• CIZ1b is a promising blood biomarker for early detection of lung cancer.
• CIZ1b biomarker is a CIZ1 fragment that spans a cancer-specific exon boundary.
• CIZ1b fragment is complexed with fibrinogen alpha chain in cancer patient plasma.
• A sandwich immunoassay for complex can measure levels in cancer patient plasma.
• Stability and specificity make it suitable for routine detection of lung cancer.

## 1 Introduction

A unique protein epitope, created by alternative-splicing of the *CIZ1* gene, is present in the plasma fraction of blood from patients with lung cancer, making it a strong candidate for high impact, high throughput early detection of lung cancer. We showed previously that detection of this b-variant form of CIZ1 (CIZ1b) is sensitive enough to allow accurate identification of patients with stage 1 disease, evidenced by an archived set of cases and matched high-risk controls, including benign lung nodules, inflammatory diseases of the lung, asthma, COPD and age-matched smokers [1]. Biomarker detection is reproducible and quantifiable in less than a microliter of plasma by western blot, giving very favourable outputs in two independent archived cohorts, with 170 and 160 samples respectively. For set 1, mean CIZ1b level in individuals without diagnosed tumours established a threshold that correctly classified 98% of small cell lung cancers (SCLC) and non-SCLC patients (receiver operator characteristic AUC 0.958). Within set 2, comparison of stage 1 non-SCLC with asymptomatic age-matched smokers, or individuals with benign lung nodules, correctly classified 95% of patients (AUCs 0.913, 0.905), with overall specificity of 76%, and 71% respectively. Moreover, using the mean of controls in set 1, we achieved 95% sensitivity among stage 1 non-SCLC patients in set 2 with 74% specificity, demonstrating the robustness of the classification. Thus, CIZ1b performs with clinically useful accuracy, and could potentially satisfy the unmet need for a circulating biomarker for early detection of lung cancer.

However, clinical development of this promising blood test requires translation of western blot-based analysis of denatured samples, into a high-throughput immunoassay format, compatible with near native epitopes. The unusual and complicated composition of the biomarker has limited timely translation to immunoassay format, however based on detailed analysis of its identity in lung cancer patient plasma we now report development of a quantitative sandwich ELISA. We also report underpinning information on biomarker stability, and unexpected findings about the native format of CIZ1b, and its interaction partners in human blood. The data presented here defines the CIZ1b plasma biomarker as a small CIZ1 fragment spanning the exon 14b/15 junction stably complexed with fibrinogen alpha chain, and has lead to proof of concept validation of a quantitative sandwich ELISA that can detect the CIZ1b/fibrinogen alpha complex in lung cancer patients, via a simple immunoassay of blood.

In intact cells, the CIZ1 protein is part of the nuclear matrix (NM), a biochemically defined fraction that resists extraction from the nucleus and which is thought to play a role in spatially organising nuclear process such as DNA replication [2]. CIZ1 plays a role in DNA replication via a mechanism that involves tethering regulators of DNA replication, including cyclins, to the NM [3], [4]. Since it was first identified [5], CIZ1 has been linked with neurological disorders including Alzheimer's disease [6] and cervical dystonia [7], and a rapidly growing range of cancers including the pediatric tumours medulloblastoma [8] and Ewing's tumour [9], and more common adult onset cancers including breast [10], lung [1], colon [11], prostate [12] and liver cancers [13], in some cases showing promise as independent prognostic [11] or diagnostic indicators [1], and in others implicated in the proliferation characteristics of cultured cancer cells [14], [15]. Notably, CIZ1 transcripts are alternatively spliced to yield 25 (Aceview 2012) or more [16] variants. Most are not fully characterised but some, including CIZ1b, appear to be inaccurate splicing events expressed only in tumours [1], while other ‘normal’ variants are associated with disease when expressed in the wrong developmental context [9], [17]. Our previous functional analysis of CIZ1 informed our understanding of the altered forms detected in lung cancer libraries, and led us to focus on CIZ1b, and then to identify CIZ1b protein in blood plasma. However, while we now understand much about CIZ1s function inside the nucleus, we do not yet know whether circulating CIZ1b has biological significance in the blood, other than as a very potent marker of malignant lung tumours.

## 2 Results

### 2.1 Biomarker characterisation

**Fig. 1:**
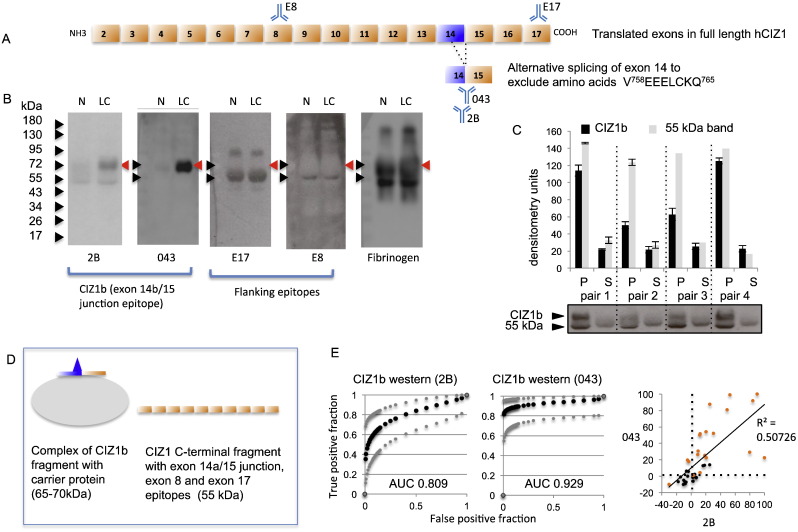
Exon junction specific antibodies detect CIZ1b in lung cancer patient plasma. A) Map of *CIZ1* translated exons showing alternative splicing of exon 14 to yield 14b (CIZ1b) by exclusion of the indicated 8 amino-acids (positions refer to NCBI Reference Sequence: NM_012127.2). Also shown are the location of epitopes detected with antibodies used in B. B) Western blot of representative plasma samples from a patient with NSCLC (LC) and normal control (N) after denaturation for SDS-PAGE, showing CIZ1b band at 65–70 kDa in LC (red arrow) detected by CIZ1b antibodies 2B and 043, and a ‘generic’ CIZ1 antibody-reactive band at 55 kDa in both N and LC, detected via epitopes in exon 8 and exon 17. Also indicated is fibrinogen alpha chain. C) Comparison of CIZ1b levels in paired plasma (P) and serum (S) samples from four individuals analysed with CIZ1b antibody 2B. Histogram shows quantified band intensities for the 65–70 kDa and 55 kDa entities, derived from triplicate samples, with standard deviation. Note that serum lacks the 65–70 kDa CIZ1b band. D) Model showing the simplest interpretation of the data in B, though extensive alternative splicing and likely secondary modification mean that the exact identity of the 55 kDa entity in plasma is unverified. E) Receiver operating characteristic (ROC) curves showing area under the curve (AUC) values for 2B and 043 western blot results on lung cancer test set A. Dot plot shows correlation between the two date sets (LC samples are orange). Thresholds (black dotted lines) are set at the mean of non-cancer samples in the set, plus one SD. These yield sensitivity/specificity estimates for this set of 20 lung cancer and 20 non-cancer samples of: 2B, 85/67.5; 043, 90/77.5. Using an arbitrary threshold specified by both antibodies (see SI Data set 1) these are 90/87.5.

Published analysis of CIZ1b was carried out with anti-peptide rabbit polyclonal antibody 2B, which was raised against a short unique peptide spanning the alternatively spliced 14b-15 junction (Fig. 1A), with sequence DEEEIEVRSRDIS (junction indicated by underlined valine). The strategy for generation, validation and purification was described previously [1]. The western blot reactivity profile for 2B includes a 65–70 kDa entity in SDS-PAGE of plasma from lung cancer patients, plus a 55 kDa entity in plasma from all people (Fig. 1B, and Fig. S1A). The cancer-specific 65–70 kDa species is the CIZ1b biomarker that is referred to here and previously [1]. Crucially, unlike plasma, we were unable to detect this protein in serum samples from the same lung cancer patients (Fig. 1C), suggesting that CIZ1b biomarker is sequestered in coagulated blood.

Notably, the 55 kDa generic band co-migrates with CIZ1 species detected with anti-CIZ1 exon 17 antibodies, and an anti-CIZ1 exon 8 antibody (Fig. 1B), identifying the 55 kDa species as a variant or proteolytic fragment of full-length hCIZ1 (which has a predicted full length MW of 99 kDa, NCBI Reference Sequence: NM_012127.2). The width of the bands detected via exon 8 and exon 17 is different, possibly because the exon 8 epitope lies within an alternatively spiced region, while detection via exon 17 would reveal species with or without alternative splicing at exon 8. Thus, at least one form of the CIZ1 protein is present in the circulatory system in cancer and non-cancer plasma samples alike, suggesting it to be part of normal physiology.

Exon 17 and exon 8 epitopes are not present in the 65–70 kDa species recognised by CIZ1b antibody 2B in cancer patients, which means that sequences both upstream and downstream of the CIZ1b epitope are missing from this protein, raising questions about its identity. The data suggest that the 65–70 kDa species is either a complicated CIZ1 splice variant, or an SDS-stable complex between a CIZ1b fragment of CIZ1 and a carrier protein (Fig. 1D).

In order to understand the relationship between cancer-specific 65–70 and generic 55, and to ask whether 55 i) incorporates the 14b/15 junction or ii) is detected by 2B via epitopes that immediately flank the junction, we generated a further set of rabbit antibodies using the same immunisation protocol as for 2B. This yielded antibody 043, which cleanly and uniquely reacts with the 65–70 kDa band in cancer patients (Fig. 1B, Fig. S1A, B). Thus 043 is a much cleaner detection tool than 2B with potential for application in other assay formats, including ELISA. Lack of recognition of the 55 kDa species suggests less contribution of junction flanking sequences to its epitope compared to 2B, and suggests that the 55 kDa species does not contain the CIZ1b junction.

Direct comparison of the ability of these two antibodies to discriminate lung cancer patients by western blot shows them both to be discriminatory, with a high degree of correlation (Fig. 1E), though notably they do not share exactly the same profile across plasma test set A. Thus, there is some variation in their epitopes but a common ability to discriminate patients with lung cancer from those without. There is also a small advantage in integrating their output to maximise selectivity (raw data in SI Data set 1).

### 2.2 Stability

The published [1] and current data support the assertion that CIZ1b is a potent circulating biomarker for lung cancer with significant potential for application in clinical practice. However to realise this potential, it must be robust enough to withstand variations in sample processing and collection regimes that occur in busy hospitals. Therefore, we tested the stability of the 65–70 kDa cancer-selective CIZ1b species in plasma and whole blood, and compared it with the 55 kDa species, using antibody 2B. The data show stability in isolated plasma over at least 6 h when incubated at 37 °C, with gradual decay in both species thereafter (Fig. S2A). Similarly, there was relatively little change during an hour at temperatures up to 50 °C (Fig. S2B), and stability over at least 3 freeze-thaw cycles (Fig. S2C). Furthermore, when whole blood was left on the bench for up to 24 h prior to fractionation there was no loss of signal (Fig. S2D), all of which indicate it to be a robust biomarker suitable for application in a range of clinical settings.

### 2.3 Complex dissociation

**Fig. 2:**
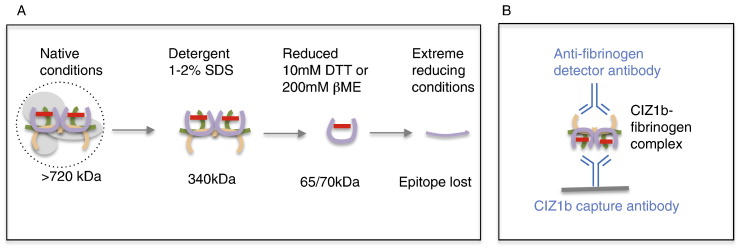
Deconstruction of CIZ1b biomarker and immunoassay design. A) Schematic of results shown in Fig.S3, illustrating the complexity of the native complex in which CIZ1b epitope (red bar) resides in plasma, and the disrupting effect of detergent and reducing agents. Under native conditions CIZ1b is part of a complex in excess of 720 kDa, most likely encompassed within lipid vesicles (dotted circle, see also Fig.S5). SDS shifts the epitope to 340 kDa, and reducing agents shift it further to the mobility typically observed in standard SDS-PAGE gels (65–70 kDa). In the presence of excess reducing agent the western blot epitope is lost for both 2B and 043 CIZ1b antibodies. B) Schematic of sandwich ELISA format based on information summarised in A.

Under native conditions both 2B and 043 detect a complex in excess of 720 kDa that co-migrates with an abundant protein in plasma (shown for 043 in Fig. S3A), but there is very little discrimination between cancer and non-cancer plasmas. Upon further separation through a second dimension under denaturing conditions the cancer-specific 65–70 kDa species is revealed and (for 2B) separated from the 55 kDa generic band (Fig. S3B). Thus, cancer-specific detection of CIZ1b in a completely native immunoassay format is unlikely even with 043. We therefore dissected out the three major denaturing influences (heat, SDS, reducing agent) that reveal cancer-specific signal and evaluated their effect on the mobility and discrimination of CIZ1b by 043. This showed that 1% SDS shifts the major reactive species from > 720 kDa in native gel, to approximately 340 kDa (Fig. S3C, right), revealing cancer-specific signal, as well as an unspecific band at ~ 200 kDa in both cancer and non-cancer samples. Increasing the incubation temperature has a detrimental effect on the cancer-specific species, with maximum differential achieved at 37 °C. Inclusion of reducing agent further shifted the mobility of the cancer-specific signal to the expected position of 65–70 kDa (Fig. S3C, left). Notably, higher concentrations of reducing agent resulted in loss of CIZ1b signal (Fig. S3D,E). Taken together, these data suggest that the 65–70 kDa entity is itself a complex that is resistant to standard SDS-PAGE denaturing conditions (10 mM DTT) but not more aggressive treatments (500 mM DTT), and that it is normally present within a higher order complex of 340 kDa, which is itself released by SDS from a still bigger complex in excess of 720 kDa (illustrated in Fig. 2A). In fact, fractionation of plasma to enrich for the exosomal compartment differentially partitioned the 55 kDa species (detected via exon 17) and 65–70 kDa species (detected via 043), with the 65–70 kDa species detected exclusively in the pellet, which is a complex fraction that includes exosomes (Fig. S4A,B).

### 2.4 Identification

**Fig. 3:**
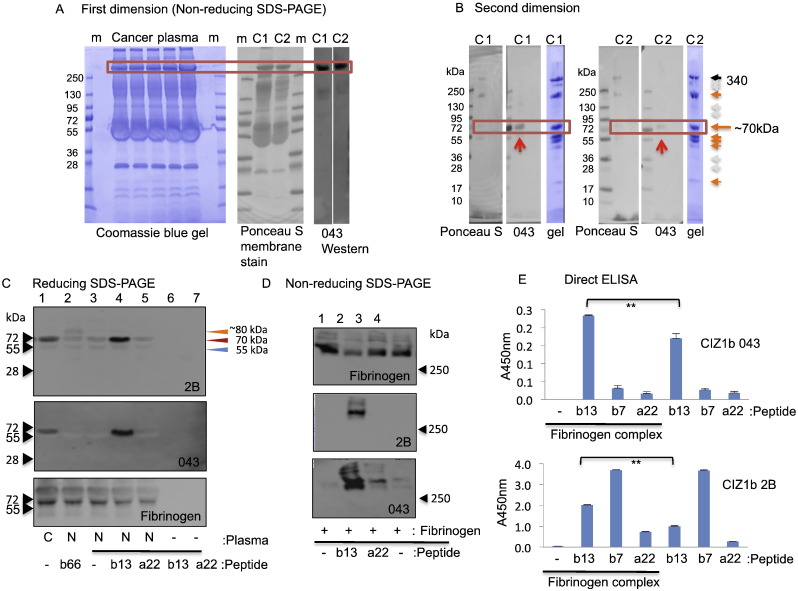
Reconstitution of CIZ1b biomarker from purified components. A) Two independent lung cancer patient plasmas were separated after incubation with 1% SDS (no reducing agent), in order to isolate the 340 kDa band from stained gels (left). Band identity was verified by Ponceau S stain followed by western blot with anti-CIZ1b antibody 043 of a parallel gel (right). B) Gel slices were soaked in 2% SDS-PAGE loading buffer (with 200 mM βME) and separated through a denaturing gel to recover the 043-reactive species at 70 kDa. Note that sample loaded as a gel slice is slightly retardation compared to markers which are loaded in solution. Coomassie blue staining revealed 5 dominant bands including one at 70 kDa, which was isolated and digested with either trypsin (cancer plasma 1, C1) or AspN (cancer plasma 2, C2). C) Western blot of normal human plasma (non-cancer, N) showing reconstitution of CIZ1b epitope via complex formation between a carrier protein in plasma and synthetic CIZ1b peptides of the indicated lengths, but not equivalent CIZ1a peptide (Table S1). Note that free peptides migrate in reducing SDS-PAGE with a relative mobility 3 times expected (~ 21 kDa instead of 7.6 kDa for b66, ~ 5 kDa instead of 1.6 for b13, see Fig. S4C). None are recognised by CIZ1b antibodies in western blot unless complexed with carrier protein. For CIZ1b66 peptide a new reactive species is created at ~ 80 kDa (see also Fig. S4C), while CIZ1b13 peptide increases antigenicity at the same mobility as the endogenous epitope in lung cancer plasmas (C, lane 1). D) Western blot of non-reducing SDS-PAGE gel showing purified fibrinogen (6 nmol) in all lanes (upper), with and without prior incubation with 100 pmol of the indicated peptides (Table S1), probed with CIZ1b antibodies as indicated. Under non-reducing conditions fibrinogen migrates with apparent molecular weight of ~ 340 kDa and this co-migrates with CIZ1b signal. E) Direct ELISA showing mean A450 nm (triplicate analysis with SEM) generated by CIZ1b antibodies 2B or 043 as indicated, using molar equivalents of preformed peptide/fibrinogen complex as analyte, as described in Methods. **t-test *P* < 0.05.

These observations enable a two-step gel-purification strategy for the 65–70 kDa CIZ1b band. First, the 340 kDa species was isolated from two independent lung cancer patient plasmas (Fig. 3A), followed by further separation in the presence of reducing agent (Fig. 3B). The 65–70 kDa band corresponding to the cancer-specific western blot signal was excised and digested with either trypsin or Asp-N endoprotease, for identification by mass spectrometry (MS). For both enzymatic digestions and two patients, Mascot database searching returned a single significant identification (*P* < 0.05), corresponding to Uniprot P02671; human fibrinogen alpha chain. The endogenous fibrinogen molecule is a hexameric glycoprotein with a pre-cleavage formula mass of 326 kDa and relative mobility of ~ 340 kDa, comprised of two sets of three different chains (α, β, and γ) of 67, 51 and 45 kDa respectively [18].

Notably, CIZ1b was not identified in the 65–70 kDa band after digestion with either enzyme. To determine whether a diagnostic CIZ1b fragment could be observed by MS if present, a short synthetic CIZ1b peptide (b13) and long CIZ1b peptide (b66, Table S1) were digested with Asp-N, which generates the diagnostic fragment DEEEIEVRSR by cutting either side of the CIZ1b exon junction (Fig. S5A). The peptide was positively identified post digestion of b13 and b66 by MALDI-MS/MS, though at low intensity relative to other peptides in the digest (shown for b66 in Fig. S5B). Further analysis was performed by LC-MS/MS using both data dependent acquisition (DDA) and targeted selection reaction monitoring (SRM), which focused the analysis to cycle through fragmentation of the expected 2^+^ and 3^+^ ions of DEEEIEVRSR, excluding all other precursors, to maximise specificity and sensitivity for this analyte. DEEEIEVRSR was identified in both the DDA and SRM analyses, indicating that the diagnostic peptide is observable by MS when excised from chemically synthesized peptide (SRM chromatogram, Fig. S5C). However, when the same peptide was spiked into normal human plasma to reconstitute CIZ1b (see later and Fig. S5D), the diagnostic DEEEIEVRSR peptide was not detected in the excised 65–70 kDa band (SRM chromatogram, Fig. S5C). Lack of identification could reflect: i) secondary modifications that alter the expected mass of the CIZ1b diagnostic peptide or impair enzymatic digestion, ii) the more abundant proteins in the sample, primarily fibrinogen alpha chain, causing ion suppression or iii) absolute abundance falling below the limit of detection for the instrumentation after the losses incurred from the PAGE and digestion steps. It should be noted that the peptide is highly acidic, which is likely to reduce ionization efficiency in positive-mode MS, so that even with SRM a small drop in abundance could fall below the limit of detection. We concluded that if we cannot reliably detect the spiked peptide at physiologically relevant levels we are unlikely to detect endogenous CIZ1b, and so followed alternative approaches.

### 2.5 Epitope reconstitution

In order to confirm that the 65–70 kDa cancer-specific epitope does indeed contain CIZ1b sequences, we adopted an epitope reconstitution strategy. To achieve this a pair of synthetic peptides spanning the exon14/15 junction (CIZ1b13, and CIZ1a22 which includes the alternatively spliced intervening sequence, Table S1) were incubated with normal human plasma (Fig. 3C), and the output compared to lung cancer patient plasma. The CIZ1b peptide, but not CIZ1a, generated 043 and 2B-reactive epitope of comparable mobility to the endogenous epitope (65–70 kDa), which is far in excess of the relative molecular mass of free peptide and not present in either of the individual components of the reaction (peptide lanes 6, 7, or plasma lane 3). This suggests that CIZ1b peptide forms an SDS/DTT-resistant complex with a carrier protein in human plasma, recreating the CIZ1b biomarker recognised by 2B and 043. Since we did not detect a mobility difference between endogenous (cancer plasma lane 1) and reconstituted epitope, the data also imply that the CIZ1b fragment contributing to the 65–70 kDa cancer-specific species is very short, in the order of 10–20 amino acids in length. Furthermore, individual molecules may not necessarily have uniform molecular boundaries, possibly accounting for the often-diffuse nature of the band (Fig. S1A).

A molar equivalent of the much longer CIZ1b peptide (b66, Table S1) reconstituted reactive epitope less efficiently, and was detectable only by 2B (Fig. 3C lane 2, and at higher concentration in Fig. S5D). With this peptide the reconstituted signal migrated with greater relative molecular mass, at ~ 80 kDa, consistent with the increased size of the contributing peptide. Importantly, reconstituted CIZ1b epitope, like the endogenous epitope, resists standard denaturing SDS-PAGE conditions (10 mM DTT, 2% SDS, 90 °C), but is dissociated by a more aggressive reducing environment (Fig. S3E). Taken together, the data suggest that, *in vivo*, the cancer-specific epitope is composed of a relatively short fragment of CIZ1 encompassing the exon14b/15 junction, mounted on fibrinogen alpha chain, which itself makes up most of the mass of the 65–70 kDa species. To further confirm this, epitope reconstitution experiments were performed with purified human fibrinogen (Fig. 3D). For both antibodies, reactive epitope was generated at the mobility of fibrinogen complex (340 kDa in non-reducing gels) and the specificity of the signal was confirmed by lack of reactivity with equivalent CIZ1a peptide. Direct ELISA using human fibrinogen complexed with molar equivalents of CIZ1a and CIZ1b peptides (Fig. 3E) confirmed three things; i) the specificity of epitope reconstitution with peptide b13 over a22, ii) negligible signal with fibrinogen alone, and iii) greater reactivity when peptide is mounted on fibrinogen. Moreover, inclusion of the very short CIZ1b peptide b7 (Table S1) revealed differences between 2B and 043; 2B recognises b7 while 043 does not in a manner that is unrelated to the presence of fibrinogen. The existence of CIZ1b epitope in a complex with fibrinogen is consistent with our earlier observations that the biomarker is depleted from serum samples isolated from clotted blood (Fig. 1C). Thus, future clinical evaluation and application of this biomarker must avoid serum samples, and ensure stable use of anticoagulants.

### 2.6 CIZ1b immunoassay

**Fig. 4:**
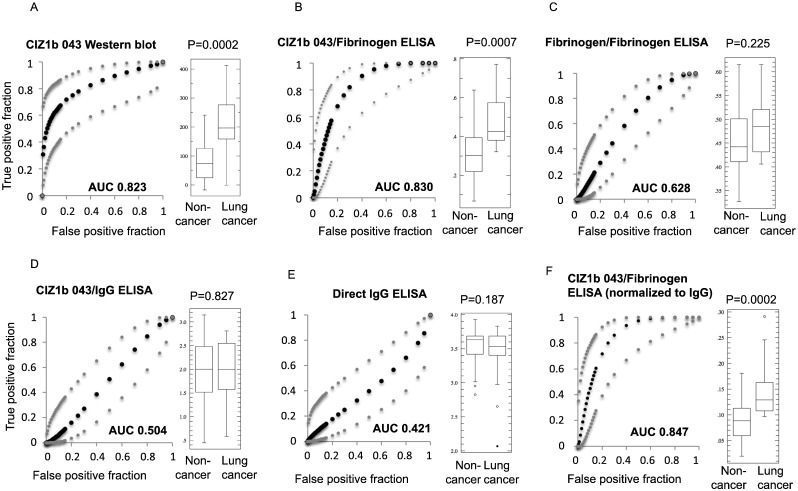
Immunoassay validation using lung cancer plasma and controls in development set B (*n* = 39 non-cancer, 13 lung cancer). A) CIZ1b western blot with antibody 043. B) Sandwich ELISA format for CIZ1b antigen in plasma, using 043 capture antibody and anti-fibrinogen detector antibody. Comparison of the two data sets is in Fig. S6B. C) Quantitative detection of fibrinogen in the same samples using paired fibrinogen antibodies (calibration is in Fig. S6C). D) Substitution of anti-human IgG for anti-fibrinogen in a sandwich format similar to that shown in B. E) Detection of IgG in plasma by direct ELISA. F) Normalization of the data in C against the data in G. In all cases, receiver operating characteristic curves (ROC) indicate the relationship between true positive and false positive fraction (black points) and 95% confidence interval of the fitted ROC curve (grey points), and area under the curve (AUC) values in bold. Box and whisker plots display minimum and maximum, lower, median, and upper quartile, and outliers.

Based on knowledge of the composition of CIZ1b biomarker, we designed a sandwich immunoassay format capable of quantitative detection in partially denatured plasma. Antigen capture with CIZ1b antibody 043 from 5 μl of plasma (Fig. S6A) and detection via fibrinogen alpha chain, generated a cancer-specific signal, illustrated with assay development set B, described in Methods. Specifically, results by western blot with 043 generated ROC AUC 0.823 (*P* = 0.0002) and by ELISA with 043/anti-fibrinogen of 0.830 (*P* = 0.0007, Fig. 4A,B). Thus, the two methods generate very similar outcomes, and the data is significantly correlated (Fig. S6B).

Set B, which represents patients with a wide range of conditions presenting at York Hospital respiratory medicine clinics, was also used to evaluate the contribution of the CIZ1b component of the assay, since fibrinogen alone has been reported to have some potential as a biomarker for lung cancer [19]. Quantitative detection of fibrinogen in the linear range (Fig. S6C,D) returned some discriminatory capability across this set, though relatively poor ROC AUC of 0.628 (*P* = 0.225). This is consistent with western blot evaluation of fibrinogen (Fig. S1B), which indicated little difference between lung cancer patients and those without malignant disease. Thus capture of CIZ1b is the element that contributes the majority of the cancer-selectivity to this assay. We further tested the specificity of the configuration by analysing Set B after capture with 043, but substituting anti-fibrinogen detector antibody with anti-human IgG (Fig. 4D). Results indicate no discrimination between patients and control plasmas, confirming that anti-fibrinogen is an informative detector reagent that specifically detects a cancer-selective complex retrieved via CIZ1b. Finally, measurement of total IgG levels across set B by direct ELISA (Fig. 4E) was used to normalize the output generated by 043/fibrinogen, leading to a marginal improvement in discrimination and ROC AUC 0.845 (*P* = 0.0002, Fig. 4F). Thus, we report here a straightforward sandwich ELISA format suitable for high-throughput application on hospital platforms, that can reliably quantify circulating CIZ1b biomarker, and which can be used to identify patients with early stage lung cancer.

## 3 Discussion

Primary lung cancer remains the main cause of cancer death (1.3 million pa worldwide). Early detection of lung cancer is important because the only curative therapy today is surgical resection of early-stage tumours [20]. Crucially, for patients whose lung cancers are detected at stage 1 the outcome is substantially better than average. For stage 1A non-small cell lung cancer (NSCLC), the 5-year survival is 58–73% and for stage 1B 43–58%. However, NSCLC is usually diagnosed later when outcomes are significantly worse (stage 2A 36–46%, stage 2B 25–36%, stage 3A 19–24%, stage 3B 7–9%, stage 4 2–13% [21]). Screening programs using chest X-ray (CXR) or low-dose computed tomography (LDCT) can identify the disease at an early stage, and therefore have the potential to improve survival. The largest study concluded that a significant reduction (20%) in mortality can be achieved among high-risk populations by regular screening of pre-symptomatic individuals, however around a quarter of those screened were referred for invasive follow-up procedures to investigate suspicious imaging results, and around 96% of these turned out to be false positive [22]. Similar findings were made by an independent trial using a population-based questionnaire to identify high-risk individuals [23]. Thus both the personal and economic costs of screening make stand-alone imaging strategies difficult to justify. Further refined patient selection criteria coupled with a non-invasive technology that can eliminate a substantial proportion of false positive results, could add impact to early detection initiatives, by reducing over-diagnosis and at the same time reducing demand for LDCT.

Two potential technologies that could meet this need are breath analysis [24] and circulating biomarkers. A number of studies suggest candidate biomarkers, usually panels of tumour proteins [25], [26] or autoantibodies [27], and an associated risk index. There is also much exploration of circulating DNA, RNA and methylation [28]. However, we know of no other publicly available data set that evaluates a single analyte blood-based biomarker on cohorts with stage 1 disease, with the degree of accuracy and stability seen for the CIZ1b biomarker [1]. Furthermore, to-date none of the candidates have been validated on prospectively collected cohorts, or directly compared to LDCT. The present technology will facilitate such comparison for CIZ1b, by enabling high-throughput measurement on a hospital-friendly platform.

Clinical biomarkers have been instrumental in reducing mortality in cancer patients [29], however lack of understanding of the capability of biomarker tests can result in inappropriate use and lead to over-investigation of disease that is incorrectly assumed to be malignant [30]. Furthermore the value of some has been questioned especially in relation to their use in the initial diagnosis of cancer [29], [31]. Lack of clarity usually arises when a marker is found in other pathologies or when, like PSA, it is not specific for neoplastic cells. While further investigation will be required for CIZ1b we already know that it can distinguish between early stage malignant disease and non-malignant conditions that may appear similar at presentation [1].

The CIZ1b epitope that we measure in the blood may be the residue of degraded circulating tumour cells, or it may be a biologically active form that is secreted by cells. In support of the second possibility, non-variant epitopes of CIZ1 are consistently detected in plasma from people with or without cancer indicating that presence in the blood is part of normal CIZ1 biology. Crucially, the CIZ1b form can be robustly measured in microliter quantities of plasma suggesting that it is not a rare protein in lung cancer patients. Moreover, it is an exceptionally stable analyte, which is a fundamental requirement for any biomarker in routine clinical use. By definition, nuclear matrix proteins (those that remain in the residual nucleus after extraction of chromatin, RNA, lipids and salt-sensitive proteins) are highly stable entities. CIZ1 is part of this fraction [3] though it is also detected as punctate staining in the cytosol, and in some cell types decorating the plasma membrane (unpublished).

The finding that CIZ1b epitope is part of a dramatically truncated fragment of CIZ1 that is stably associated with fibrinogen is unexpected. While it is documented that certain bacterial proteins, such as extracellular fibrinogen-binding protein (Efb) of *S. aureus* [32], can impact on fibrinogen-dependent platelet aggregation, it is not known at this point whether CIZ1b fragment has any impact on functionality, though the long CIZ1 peptides used here (b66, a74) do share some similarity with the consensus binding sequence of Efb. While a state of hypercoagulability is well-known to be associated with malignancy including primary lung cancers [33], the effect of CIZ1b remains to be investigated.

A number of retrospective studies have suggested that fibrinogen levels may offer information that can be used to identify or monitor the course of disease, for example as part of a panel of analytes involved in haemostasis and angiogenesis in breast cancer patients [34], or via measurement of fibrinogen in urine samples from bladder cancer patients [35]. Moreover, a recent study of 84,000 individuals that looked for elevation of three inflammatory biomarkers (CRP, fibrinogen and leukocyte count) observed an increased risk of colorectal, lung and breast cancer, with greatest risk associated with elevation of all three [19]. While we were not able to observe elevated fibrinogen by western blot in our cohorts, ROC AUCs for ELISA output for Set B did suggest some information content with respect to discrimination of the patients with confirmed cancer, though this was not statistically significant and is unlikely to provide sufficient discriminatory power on its own.

In summary, the data presented here and previously [1] show that CIZ1b is a strong candidate cancer-specific, single marker capable of identifying early stage lung cancer within at-risk groups, without resort to invasive procedures. The technological developments now reported bring a low-cost, minimally invasive and accurate blood test a step closer, with potential to significantly improve lung cancer survival by improving early detection rates.

## 4 Methods

Please see supplemental methods for details of plasma sets A and B, gels and sample preparation conditions, epitope reconstitution and mass spectrometry.

### 4.1 Antibodies for western blot

CIZ1b was detected using positively and negatively affinity purified anti-peptide rabbit pAb 2B [1], or newly developed reagent 043 protein A fraction. CIZ1 exon 17 was detected with a goat pAb against peptides in human exon 17 (Table S1) or NB74624, and exon 8 with NB74623 (both Novus Biologicals). Plasminogen was detected with Ab98262 (Abcam) and fibrinogen with goat pAb F8512 (Sigma) or mouse mAb 120-10068 (Novus Biologicals).

### 4.2 Enzyme linked immunosorbent assays

CIZ1b-fibrinogen alpha chain complex was detected by sandwich ELISA after partial denaturation of 5 μl of plasma in 100 μl of 0.5% tween20, 1% SDS in PBS, supplemented with 0.5 mM PMSF and incubation at 20 °C for 30 min with repeated vortexing. Plates (Nunc Maxisorp 442404) were coated with 1 μg of 043 CIZ1b pAb protein A fraction (Pierce 20356), overnight at 4 °C in 100 μl of 50 mM carbonate pH 9.6, then blocked for 2 h at room temperature with 300 μl of filtered 1% BSA (Jackson ImmunoResearch 1000162) in PBS. After three washes in 300 μl of 0.05% tween20 in PBS, analyte was added for 2 h at room temperature with gentle shaking. After five washes, captured complex was detected with sheep anti-fibrinogen pAb (AF4786 R&D systems) followed by anti-sheep HRP (Jackson ImmunoResearch 213-032-177), and results developed using 100 μl Sure Blue (KPL 520001) with detection at A450 nm using a FLUOstar OPTIMA plate reader (BMG Labtech). For measurement of fibrinogen capture antibody was chicken IgY (1 μg/well, Genway 15-288-22856), and analyte concentration was reduced five orders of magnitude by serial dilution. For measurement of human IgG in direct ELISA, 0.5 μl of plasma was coated directly onto plates in 100 μl of 50 mM carbonate buffer pH 9.6, blocked, washed and detected with anti-IgG (Jackson ImmunoResearch 709-035-149). For direct ELISA of synthetic analyte, 0.5 μg purified fibrinogen (Sigma F3879) complexed with 100 pmol peptide was coated onto plates in 100 μl of 50 mM carbonate pH 9.6 as above, probe with 043 or 2B and detected with anti-rabbit HRP (Jackson ImmunoResearch).

The following are the supplementary data related to this article.

SI Data set 1 Summary diagnoses, and assay output for patients in Set A. SI Data set 1

SI Data set 2 Summary diagnoses, and assay output for patients in Set B. SI Data set 2

**Fig. S1** A) Western blots showing three parallel gels of the same 10 plasma samples from lung cancer patients and two from individuals without disease (from Set B), probed with CIZ1b antibodies 2B and 043, and for CIZ1 exon 17. B) A different set of 5 plasma samples from lung cancer patients and 5 from individuals without disease (including one representative false positive sample, lane 4), probed with 043, and also for plasminogen (Ab98262 methods) and fibrinogen (F8512 methods).
**Fig. S2** Stability of CIZ1b in plasma and whole blood after various treatments, detected in western blot with anti-CIZ1b antibody 2B after reducing SDS-PAGE. Treatments were A) plasma after the indicated hours at 37 °C, B) plasma after 1 h at the indicated temperatures, C) plasma after freeze thaw cycles (− 80 °C for 5 min, followed by 20 °C for 5 min) in addition to the single freeze cycle received by all samples after isolation. Over 10 cycles *P* = 0.41 for CIZ1b band, and 0.49 after normalization to the 55 kDa band, indicating little degeneration. D) Whole blood samples in lithium heparin were left unchilled (approximately 21 °C) for the indicated times, prior to isolation of plasma and storage at − 80 °C. Comparison on the right shows no significant difference between plasma isolated immediately compared to after 24 h, showing Student's *t*-test values for the indicated number of measurements. After treatments all samples were heated to 90 °C for 10 min in E-PAGE loading buffer plus 200 mM β-mercaptoethanol, separated by 8% SDS-PAGE and quantified as described previously [1]. Results for the 65–70 kDa band with (closed circles) and without (open circles) normalization to the 55 kDa band are shown. Graphs show mean data (solid lines) from the indicated number of individual plasma samples (dotted lines) which were each analysed in triplicate, with SEM. Data is plotted relative to an untreated control sample in each case (once frozen plasma). Plasmas used in this series of experiments are detailed in SI Table 2.
**Fig. S3** Effect of denaturation on CIZ1b epitope. A) Left, Coomassie blue stained gel of a representative lung cancer (C) and normal (N) plasma sample (2 μl), separated under fully native conditions (in the absence of SDS or reducing agent). m indicates marker lanes. Middle, parallel gel after transfer to nitrocellulose stained with Ponceau S. Right, the same membrane probed with CIZ1b antibody 043. Lower panel shows the same two samples (red arrows) separated by denaturing SDS-PAGE, also probed with 043. B) Native gel first dimension was soaked in 4 × SDS-PAGE loading buffer for 30 min without heating, and further separated by size (second dimension). Western blot reveals one band at 55 kDa that is reactive with antibody 2B in the non-cancer sample, and two bands (55 and 70 kDa) in the cancer sample. Two additional cancer plasma samples, treated in the same way are shown below. C) Migration of 043-reactive bands in a representative cancer (C) and non-cancer (N) plasma sample through a non-denaturing gel, after prior incubation at the indicated temperatures, in the presence of 2% SDS, with and without reducing agent (200 mM β-mercaptoethanol, βME) as indicated. Note the shift of cancer-specific band (red box) from relative mobility of approximately 340 kDa in the absence of reducing agent, to ~ 70 kDa in the presence of reducing agent (accompanied by complete loss of the ‘generic’ band that was detected in all samples in the absence of reducing agent). D) Plasma from a lung cancer patient showing 043-reactive band after incubation with 1% SDS at 37 °C for 30 min, with the indicated concentrations of DTT, or a 10-fold excess of βME. The reactive band is indicated as it shifts from 340 kDa to 70 kDa, and is then lost under maximally reducing conditions. The behaviour of fibrinogen in the same samples is shown for comparison, detected with antibody F8512. E) Synthetic CIZ1b epitope generated by combining CIZ1b peptide (b66, SI Table 1) with normal (non-cancer) plasma (see Fig. 3), behaves similarly to endogenous epitope, dissociating from within a high molecular weight complex, to a ~ 70 kDa species, and then disappearing under maximally reducing conditions. Fibrinogen is detected with antibody AF4786. βME concentrations refer to multiples of the standard concentration used in SDS-PAGE of 200 mM.
**Fig. S4** A) Western blots of reducing SDS-PAGE gel showing CIZ1b and the exon 17-containing species in lung cancer patient plasma, after fractionation using a total exosome isolation kit (Invitrogen 4484450 methods). Lanes show equivalent of 0.5 μl of plasma before (lane 1) and after (lane 2) addition of exosome precipitation reagent, before centrifugation (Total, T). The supernatent (SN) and pellet fraction (P) after centrifugation are in lanes 3 and 4. Lanes 5 and 6 show 1 and 2 μl equivalents of the pellet fraction respectively. Note that the CIZ1b containing species partitions with the pellet fraction while the exon 17-containing species partitions primarily with the supernatant (red arrows). B) Exosome fractionation in the presence of detergent (1% SDS), and either 2 mM EDTA or protease inhibitor cocktail (Pi) as indicated. Note the detergent-dependent shift of CIZ1b into the supernatant fraction. C) Reducing SDS-PAGE gels stained with Coomassie blue to reveal mobility of purified synthetic CIZ1 peptides. Long CIZ1b peptide (1) and CIZ1a equivalent (2) migrate with apparent molecular weight approximately equivalent to three times their formula weight, at ~ 21 kDa (right panel), indicating some retention of stable structure under reducing conditions. Similarly, short CIZ1b peptide (lane 3) migrates as a trimer, while equivalent sequences bearing single carboxylated glutamic acid residues at the indicated positions (4, 5, 6) migrate as expected for monomeric peptide, at approximately 1.6 kDa (left panel).
**Fig. S5** A) Sequence of CIZ1 long CIZ1b peptide (b66 - bold) plus five amino-acids of upstream sequence, showing cleavage sites for Asp-N (red) and trypsin (blue). The Asp-N peptide that spans the CIZ1b junction is indicated in red and labeled ‘diagnostic peptide’. B) MALDI-MS spectrum of Asp-N digested CIZ1b66 peptide showing peptides confirmed by MS^2^ fragmentation. Inset, MALDI-MS/MS spectrum of *m*/*z* 1261.6 ion identified as DEEEIEVRSR. C) Selective reaction monitoring post Asp-N digest of band P (orange, see below), and diagnostic peptide standard DEEEIEVRSR (purple) monitoring the position of the expected 2^+^ and 3^+^ ions of DEEEIEVRSR; *m*/*z* 631.3 and 421.2060 respectively. The top two panes show the extracted ion chromatograms (EICs) of the *m*/*z* 631.3. The lower two panes display the MS^1^ spectrum centered around the *m*/*z* of the 2^+^ ion at the apex of the EIC in the standard (36.6 min). The expected *m*/*z* of the peptide 631.3 is clearly visible in the standard (purple) but not present above the noise in the sample derived from band P (orange). D) Denaturing SDS-PAGE of the indicated plasma/peptide mixtures, stained with Coomassie blue, before and after isolation of the indicated band ‘P’ (orange box), containing the reconstituted CIZ1b epitope. Below, CIZ1b western blot of the same samples, showing composite epitope generated by addition of long CIZ1b peptide b66 to normal plasma (lane 2), and endogenous CIZ1b band in lung cancer plasma (lane 4). Pure peptide (lane 1) migrates with three times the expected molecular mass (~ 21 kDa, Supplemental Fig. 4C) and is not detected by the CIZ1b antibody until complexed with carrier protein (lane 2). Note, that epitope reconstituted from peptide b66 migrates at 82 kDa, which is larger than either endogenous epitope or that created by short CIZ1b peptide b13 (see Fig. 3). Right, Ponceau S stained membrane.
**Fig. S6** ELISA optimization experiments. A) Optimal plasma concentration for discrimination of cancer and non-cancer samples via CIZ1b/fibrinogen alpha complex ELISA, is achieved with 5 μl of plasma (YH 55 and 68) and unless stated otherwise is used throughout. B) Dot blot comparison showing the degree of similarity between western blot and ELISA data for development set B (R^2^ = 0.3575). After transposition of ELISA data to the same scale as western blot data by scaling to the same normal and cancer calibrator samples, paired *t*-test returned *P* = 0.501. C) Optimization of plasma concentration for quantitative detection of fibrinogen, showing ELISA output generated by the indicated concentrations of plasma. Methodology was as described except that SDS was not added to analyte buffer, and the CIZ1b capture antibody was substituted by 1 μg of chick anti-fibrinogen antibody (filled circles), or no capture antibody (open circles). Detector antibody was sheep anti-fibrinogen AF4786 used as described in Methods. Fibrinogen signal shows that output in the linear range is observed between 0.05 and 0.5 nl of plasma. D) Effect of the CIZ1b component on ELISA reproducibility and selectivity for cancer plasma. Graph shows mean signal from two biological and two technical replicate analyses of plasma mixtures of the indicated composition, after subtraction of no plasma control readings, with SEM. Fibrinogen measurements are carried out on the same sample mixtures as CIZ1b/fibrinogen alpha complex, after dilution to accommodate the high level of fibrinogen in human plasma. Fibrinogen is detected with high sensitivity but poor reproducibility, and little quantifiable difference between lung cancer and non-cancer plasmas, while CIZ1b signal reflects the contribution of the cancer plasma to the analyte mixture. E) Limit of detection of CIZ1b by 043 measured using the indicated concentration range of purified fibrinogen with and without complexing with peptide b13 (Table S1), as indicated. CIZ1b signal is first evident above control in the presence of approximately 1 pmol of peptide epitope, and yields a quantitative response up to and beyond 300 pmol. Grey mask highlights region expanded to right. Image 1

Supplementary material Image 2

## Conflict of interest

This work was carried out at the University of York by University staff supported by a research contract with Cizzle Biotech. Cizzle is a spin out from the University of York funded by Yorkshire Cancer Research. DC is a minority shareholder in Cizzle Biotech.

## Author contributions

GH, DW, OTJ, AH, EA, RC, AD and DC performed the research. DC designed the research and wrote the paper, with input from AD, and OTJ. DW, OTJ and JW collected human plasma samples.

## Funders

This work was funded by Cizzle Biotech (CizzleRC13-16) with additional support from the Wellcome Trust through C2D2 at the University of York (ISSF award 097829/Z/11/A) and the Jean Shanks Foundation (HYMS2011).
