# Supplementary material for: A quantitative immunoassay for lung cancer biomarker CIZ1b in patient plasma
Source: Clin Biochem. 2017 Apr;50(6):336–43. doi: 10.1016/j.clinbiochem.2016.11.015 (PMC5441127; doi:10.1016/j.clinbiochem.2016.11.015)

Supplemental Fig. 1

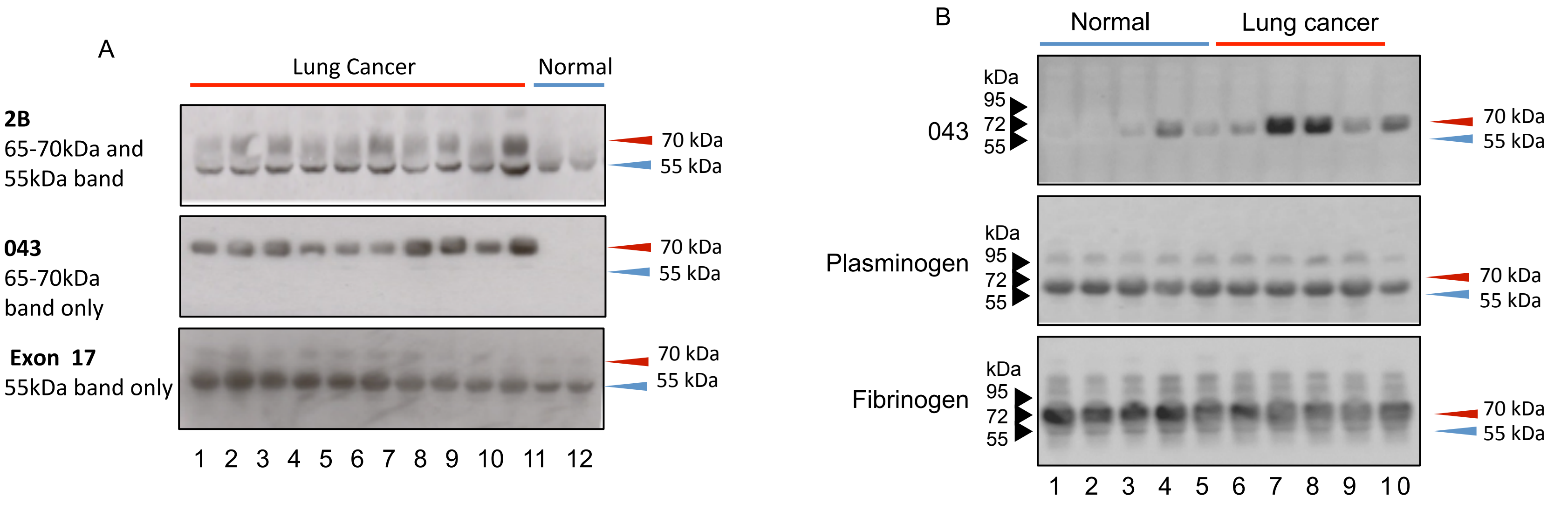

Supplemental Fig. 2

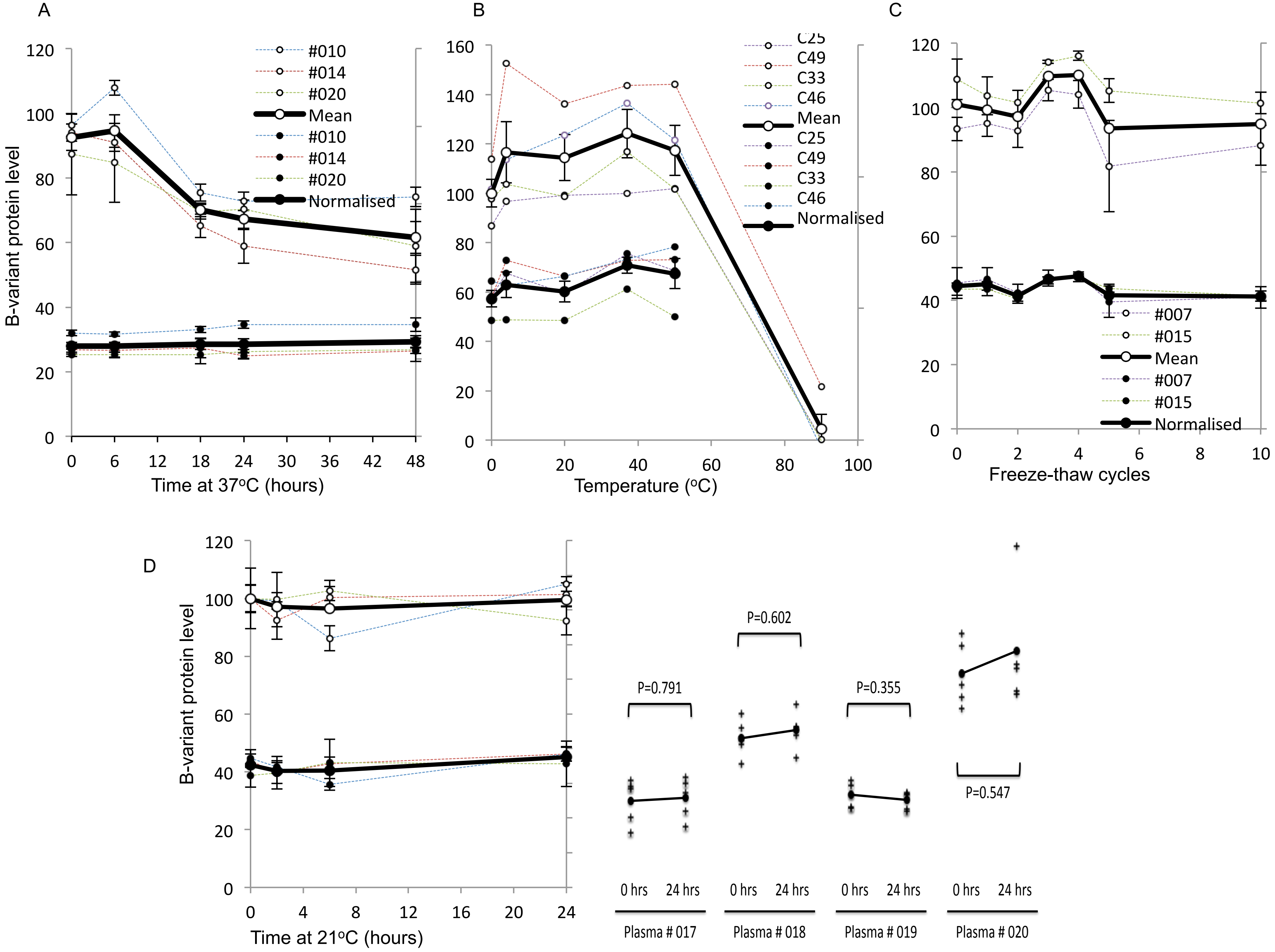

Supplemental Fig. 3

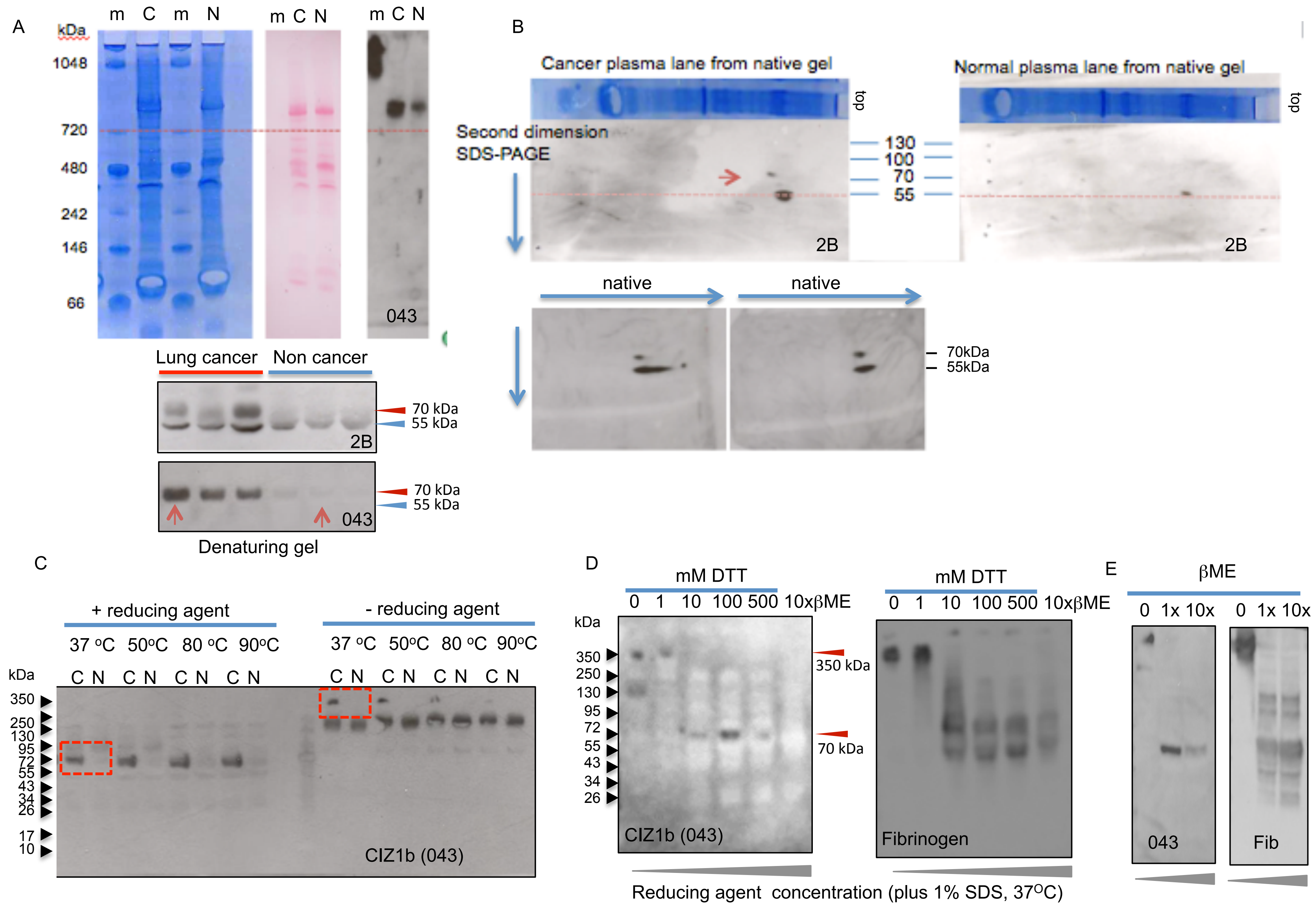

Supplemental Fig. 4

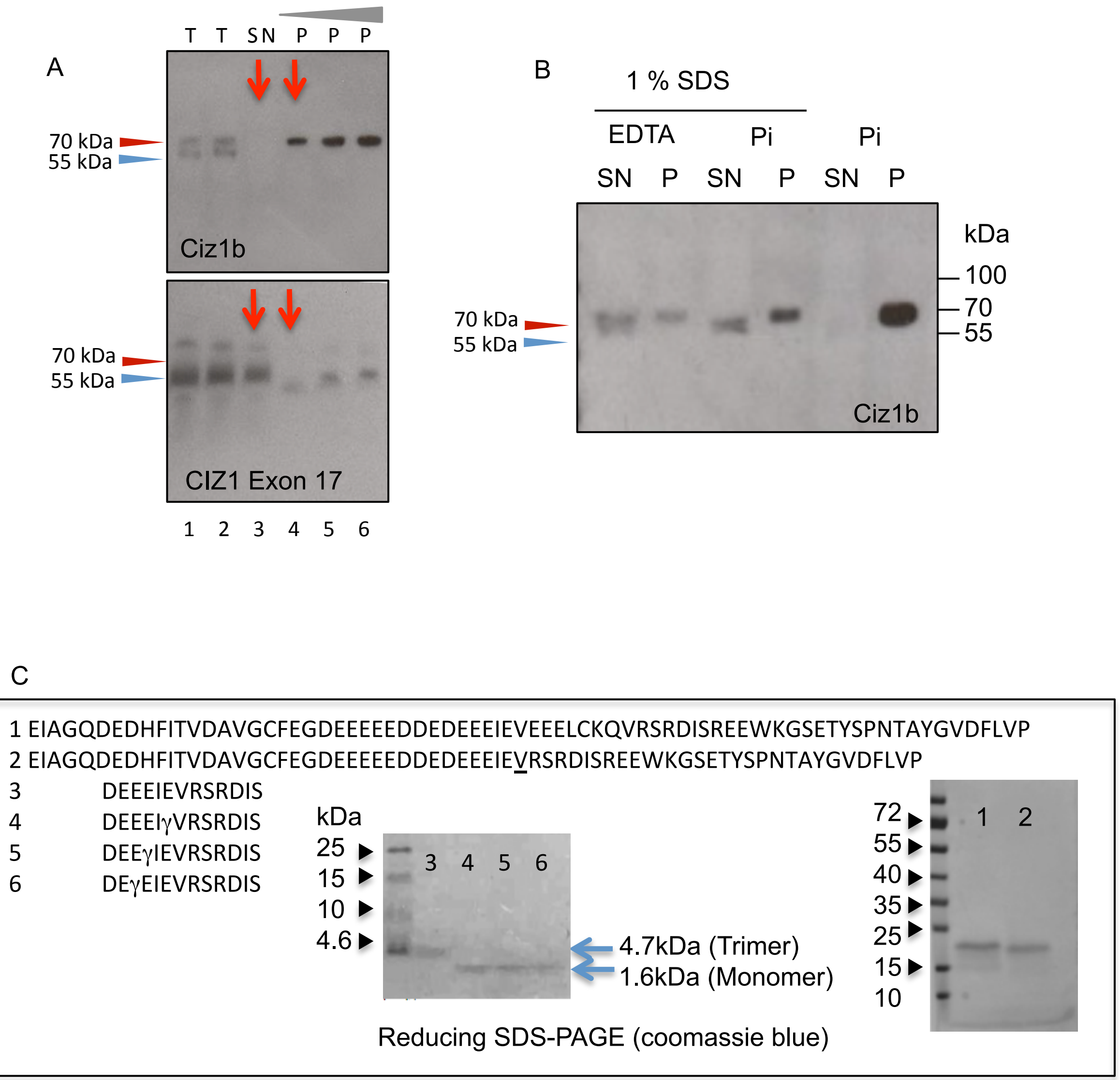

Supplemental Fig. 5

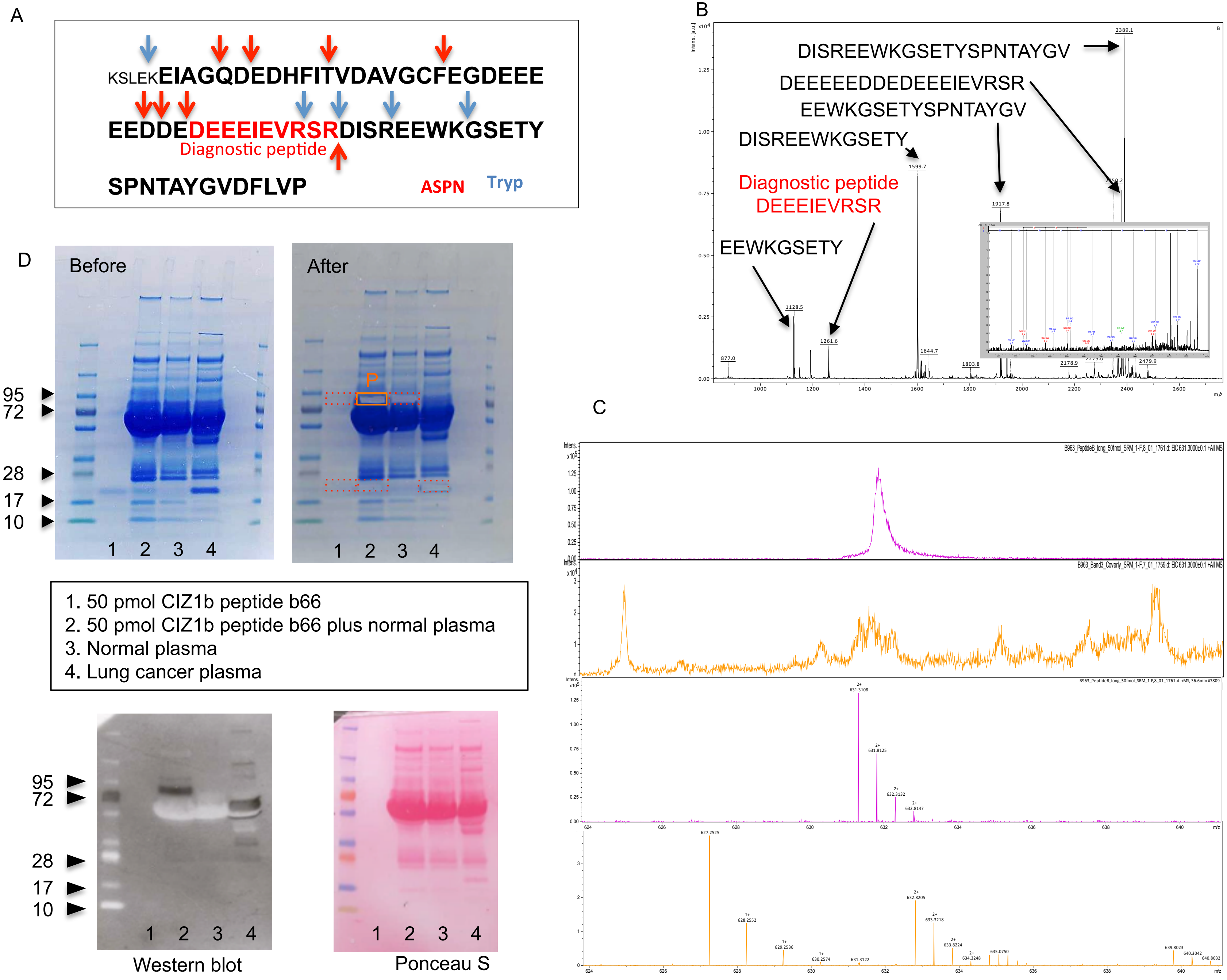

Supplemental Fig. 6

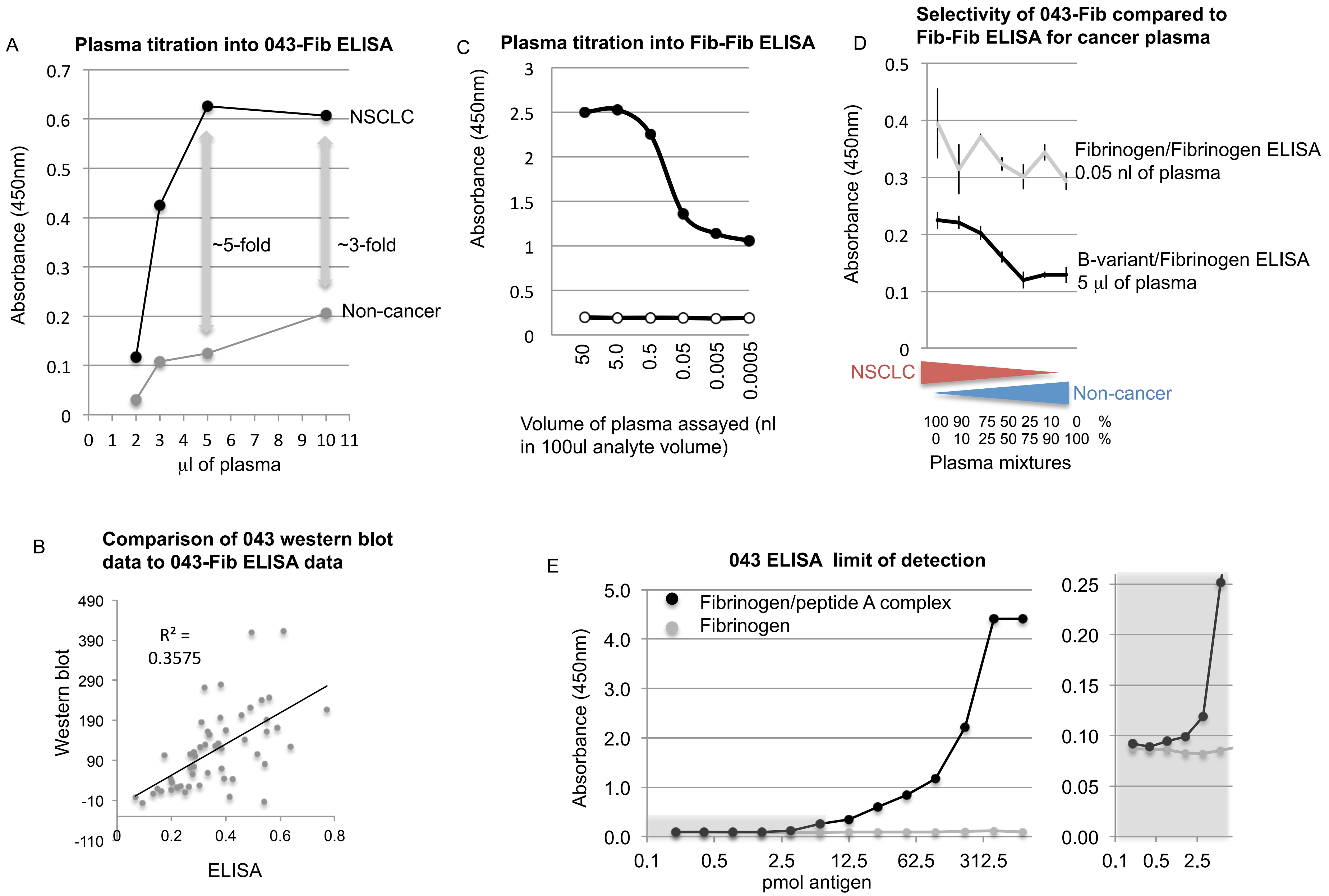

Supplement: Supplementary file 3 — Fig. S1 A) Western blots showing three parallel gels of the same 10 plasma samples from lung cancer patients and two from individuals without disease (from Set B), probed with CIZ1b antibodies 2B and 043, and for CIZ1 exon 17. B) A different set of 5 plasma samples from lung cancer patients and 5 from individuals without disease (including one representative false positive sample, lane 4), probed with 043, and also for plasminogen (Ab98262 methods) and fibrinogen (F8512 methods). Fig. S2 Stability of CIZ1b in plasma and whole blood after various treatments, detected in western blot with anti-CIZ1b antibody 2B after reducing SDS-PAGE. Treatments were A) plasma after the indicated hours at 37 °C, B) plasma after 1 h at the indicated temperatures, C) plasma after freeze thaw cycles (− 80 °C for 5 min, followed by 20 °C for 5 min) in addition to the single freeze cycle received by all samples after isolation. Over 10 cycles P = 0.41 for CIZ1b band, and 0.49 after normalization to the 55 kDa band, indicating little degeneration. D) Whole blood samples in lithium heparin were left unchilled (approximately 21 °C) for the indicated times, prior to isolation of plasma and storage at − 80 °C. Comparison on the right shows no significant difference between plasma isolated immediately compared to after 24 h, showing Student's t-test values for the indicated number of measurements. After treatments all samples were heated to 90 °C for 10 min in E-PAGE loading buffer plus 200 mM β-mercaptoethanol, separated by 8% SDS-PAGE and quantified as described previously [1]. Results for the 65–70 kDa band with (closed circles) and without (open circles) normalization to the 55 kDa band are shown. Graphs show mean data (solid lines) from the indicated number of individual plasma samples (dotted lines) which were each analysed in triplicate, with SEM. Data is plotted relative to an untreated control sample in each case (once frozen plasma). Plasmas used in this series of experiments are deta [file mmc3.pdf]
