# Supplementary material for: A quantitative immunoassay for lung cancer biomarker CIZ1b in patient plasma
Source: Clin Biochem. 2017 Apr;50(6):336–43. doi: 10.1016/j.clinbiochem.2016.11.015 (PMC5441127; doi:10.1016/j.clinbiochem.2016.11.015)
Supplement: Supplementary file 4 — Supplementary material [file mmc4.docx]

**A quantitative immunoassay for lung cancer biomarker CIZ1b in patient plasma**

Dawn Coverley ^a, b^, Gillian Higgins ^a, b^, Daniel West ^a, *^, Oliver T. Jackson ^b, d^, Adam Dowle ^b^, Aidan Haslam ^b^, Eve Ainscough ^a,b, *^ Rebecca Chalkey ^a, b^ and John White ^c^

**Supplemental information**

**Additional methods**

Plasma samples

Set A (20 lung cancer and 20 non-cancer) was supplied without identification, for blinded analysis with polyclonal antibody 2B by the protocol described previously (1). Results were decoded at a second site and used to generate the data on 2B in Fig. 1E. No information on stage or grade is available for set A. Set A was also analysed with new antibody 043 to evaluate their relative performance by western blot. Summary diagnoses and results of CIZ1b analysis are given in SI Data set 1.

Set B was collected from consenting patients attending respiratory medicine clinics at York Teaching Hospitals NHS foundation Trust, under Yorkshire & Humber Research ethics approval (11/YH/0113). Samples were collected from individuals at initial consultation irrespective of eventual diagnosis, into 2.7 ml lithium heparin S-Monovettes (Sarstedt 04.1929.001) and stored at 4^o^C for a maximum of two hours before centrifugation at 2000 x g at 20^o^C for 10 minutes. Approximately 1ml of plasma was recovered, mixed thoroughly and frozen at -80^o^C in York Hospital Clinical Research Facility. Prior to analysis plasma was thawed, centrifuged a second time at 17,000 x g, 20^o^C for 10 minutes to remove any remaining particulate material, and aliquoted to avoid subsequent freeze thaw cycles. A total of 66 pre-treatment samples were collected, of which 10 were consumed by stability analyses, resulting in 52 that subsequently received unambiguous diagnosis. These include 13 patients with lung cancer (various grades and stages) and 39 without cancer (fibrosis, COPD, pneumonia, asthma). Summary diagnoses and results of CIZ1b analysis are given in SI Data set 2.

Additional lung cancer samples, including four pairs of plasma and serum isolated from the same individual at the same time, were acquired from biorepositories including Conversant, Bioserve and Proteogenix (listed in SI Table 2). Normal control bloods were acquired from Innovative Research. A pair of pooled calibrator samples from lung cancer (C) and non-cancer (N) plasmas were used for assay development and to standardize output between experiments. Samples and composition of pools is listed in SI Table 2.

Statistics Receiver operating characteristic curves were generated using a web-based calculator (Eng J. Johns Hopkins, vailable from: http://www.jrocfit.org (accessed January 2016). Box and whisker plots were generated using calculators available at www.physics.csbsju.edu/stats/ Kirkman, T.W. (1996) (accessed January 2016). Students two-tailed T-test were calculated using Excel for Mac 2011 Ver 14.0.0.

Gels and sample preparation conditions For detection of CIZ1b in denaturing SDS-PAGE, plasma samples were prepared as described previously (1), with 0.5 ul plasma volume equivalent loaded per lane. To prepare, 5ul of plasma was mixed with 95ul of EPAGE sample buffer (Invitrogen EPBUF-01) or home-made sample buffer (2% SDS, 20 % glycerol, 75 mM Tris pH 6.8, bromophenol blue) both supplemented with fresh 200 mM ß-mercaptoethanol or 10 mM DTT, and heated to 90^O^C for 10 minutes with vortexing. The concentration of reducing agent is important to the outcome of this analysis. Samples were resolved by electrophoresis through pre-cast E-PAGE 8% 48 well gels using an E-BASE (program EG, 65 minutes), and transferred to nitrocellulose using iBlot dry blotting system (all Invitrogen). Where indicated, the heating step was omitted and/or the concentration of SDS and reducing agent were varied as indicated. In such experiments, proteins were resolved in 4-15% gradient gels (Biorad 456 1085) and transferred as above. For analysis of native complex, samples were supplemented with protease inhibitor mix (Complete EDTA free (Roche 11873580001) 1 tablet in 1 ml used at 1/50 and 2mM PMSF and mixed with equal volumes of native sample buffer (Biorad 161-0738) and electrophoresed through Biorad 456 1085 gels. Prior to transfer to nitrocellulose native gels were soaked in transfer buffer (2% SDS, 25mM Tris, 200 mM Glycine pH 8.3), so complexes were exposed to detergent prior to analysis. Where indicated membranes were stained with Ponceau S (BDH 440832H) at 0.1% in 5% acetic acid, prior to western blot. Where indicated gels were stained with Safe blue (NBS biologics SB1L) or silver (ThermoFisher Scientific 24600) according to manufacturers instructions. Membranes were probed with CIZ1b specific polyclonal antibody 2B or 043 diluted in PBS, 10% milk powder, 0.1% Tween 20, and primary antibody detected with peroxidase anti-rabbit antibody (211-032-171 Jackson Immunological Research). Primary antibody was detected with peroxidase-conjugated goat anti-mouse IgG (115-035-174 Jackson Immunological research). Bands were visualized using EZ-ECL solution (Geneflow 20-500-500A/B). Output was quantified by densitometry using NIH Image J, and values assigned by adjustment to constant positive and negative calibrator samples included in all experiment.

Reconstitution of epitope For reconstitution of CIZ1b epitope, peptides were mixed with 0.5 ul plasma in the presence of 1mM PMSF (final volume 5 ul), incubated at 37^O^C for 1hr, and processed for denaturing SDS-PAGE (reducing agent at 250 mM b-mercaptoethanol). Peptides (Table S1, Cambridge peptides) were reconstituted in water at 1 mM and stored in aliquots at -80^o^C. Typically, for long CIZ1b peptide (b66) and CIZ1a equivalent (a74) 100 pmols were used per reaction, and all short peptides at 1nmol per reaction. For reconstitution using purified fibrinogen reactions contained 6nmols fibrinogen and 100 pmols peptide in a 10ul reaction volume (1xPBS, 1mM PMSF), and were incubated at 37^O^C for 1 hour, prior to addition of sample buffer and separation through 4-15% gels (Biorad 456-1085). Human fibrinogen (Sigma F3879) was reconstituted at 1mM in water (2mM fibrinogen alpha chain which is present in two copies in each fibrinogen molecule, FW 333kDa). For synthetic analyte fibrinogen was complexed with peptides at a ratio of 15:1.

**Mass spectrometry**

Enzymatic digestion Endogenous CIZ1 species were gel purified by the following two-step process. Lung cancer plasma was diluted 1 in 10 in non-reducing sample buffer (2% SDS, 20 % glycerol, 75 mM Tris pH 6.8, bromophenol blue, 1 mM PMSF) and 1 ul equivalent separated through native 4-15% gradient gels for parallel detection of total protein with Safe blue (NBS biologics SB1L), and CIZ1b by western blot. Stained protein bands corresponding to CIZ1b epitope (350 kDa) were isolated, and further separated by SDS-PAGE after soaking for 10 mins at 37^O^C in 2% SDS, 20 % glycerol, 75 mM Tris pH6.8, bromophenol blue, supplemented with fresh 250 mM β-mercaptoethanol. Stained protein bands corresponding to CIZ1b epitope (70 kDa) were isolated and subjected to in-gel digestion with trypsin or Asp-N endoproteases. Reconstituted CIZ1b species were gel purified by a single-step process, in which plasma from normal (non-cancer) individuals was mixed with long CIZ1b peptide (b66, SI Table 1) and separated by denaturing SDS-PAGE through 4-15% gradient gels. Stained bands (~80kDa) corresponding to CIZ1b epitope were cut and and subjected to in-gel digestion with trypsin or Asp-N. In-gel digestion was performed after reduction with DTE and S-carbamidomethylation with iodoacetamide. Gel pieces were washed two times with 50% (v:v) aqueous acetonitrile containing 25 mM ammonium bicarbonate, then once with acetonitrile and dried in a vacuum concentrator for 20 min. Endoproteinase Asp-N from Pseudomonas fragi mutant strain (Sigma) was dissolved in water to give a concentration of 0.1 μg/μL. Gel pieces were rehydrated by adding 2 μL of Asp-N solution, and after 10 min enough 50 mM ammonium bicarbonate solution was added to cover the gel pieces. Sequencing-grade, modified porcine trypsin (Promega) was dissolved in the 50 mM acetic acid supplied by the manufacturer, then diluted 5-fold with 25 mM ammonium bicarbonate to give a final trypsin concentration of 0.02 μg/μL. Gel pieces were rehydrated by adding 10 μL of trypsin solution, and after 10 min enough 25 mM ammonium bicarbonate solution was added to cover the gel pieces. Digests were incubated overnight at 37^o^C.

MALDI-MS/MS A 1 μL aliquot of each peptide mixture was applied to a ground steel MALDI target plate, followed immediately by an equal volume of a freshly-prepared 5 mg/mL solution of 4-hydroxy-α-cyano-cinnamic acid (Sigma) in 50% aqueous (v:v) acetonitrile containing 0.1% , trifluoroacetic acid (v:v). Positive-ion MALDI mass spectra were obtained using a Bruker ultraflex III in reflectron mode, equipped with a Nd:YAG smart beam laser. MS spectra were acquired over a range of 800-5000 *m/z*. Final mass spectra were externally calibrated against an adjacent spot containing 6 peptides (des-Arg^1^-Bradykinin, 904.681; Angiotensin I, 1296.685; Glu^1^-Fibrinopeptide B, 1750.677; ACTH (1-17 clip), 2093.086; ACTH (18-39 clip), 2465.198; ACTH (7-38 clip), 3657.929.). Monoisotopic masses were obtained using a SNAP averaging algorithm (C 4.9384, N 1.3577, O 1.4773, S 0.0417, H 7.7583) and a S/N threshold of 2. For each spot the ten strongest precursors, with a S/N greater than 30, were selected for MS/MS fragmentation. Fragmentation was performed in LIFT mode without the introduction of a collision gas. The default calibration was used for MS/MS spectra, which were baseline-subtracted and smoothed (Savitsky-Golay, width 0.15 m/z, cycles 4); monoisotopic peak detection used a SNAP averaging algorithm (C 4.9384, N 1.3577, O 1.4773, S 0.0417, H 7.7583) with a minimum S/N of 6. Bruker flexAnalysis software (version 3.3) was used to perform spectral processing and peak list generation.

LC-MS/MS Peptide mixtures were loaded onto a nanoAcquity UPLC system (Waters) equipped with a nanoAcquity Symmetry C_18_, 5 µm trap (180 µm x 20 mm Waters) and a nanoAcquity HSS T3 1.8 µm C_18_ capillary column (75 μm x 250 mm, Waters). The trap wash solvent was 0.1% (v/v) aqueous formic acid and the trapping flow rate was 10 µL/min. The trap was washed for 5 min before switching flow to the capillary column. Separation used a gradient elution of two solvents (solvent A: aqueous 0.1% (v/v) formic acid; solvent B: acetonitrile containing 0.1% (v/v) formic acid). The capillary column flow rate was 350 nL/min and the column temperature was 60°C. The gradient profile was linear 2-35% B over 20 mins. All runs then proceeded to wash with 95% solvent B for 2.5 min. The column was returned to initial conditions and re-equilibrated for 25 min before subsequent injections. The nanoLC system was interfaced with a maXis HD LC-MS/MS system (Bruker Daltonics) with CaptiveSpray ionisation source (Bruker Daltonics). Positive ESI-MS and MS/MS spectra were acquired using AutoMSMS mode. Instrument control, data acquisition and processing were performed using Compass 1.7 software (microTOF control, Hystar and DataAnalysis, Bruker Daltonics). Instrument settings were: ion spray voltage: 1,450 V, dry gas: 3 L/min, dry gas temperature 150°C, ion acquisition range: *m/z* 150-2,000, quadrupole low mass: 300 *m/z*, collision RF: 1,400 Vpp and transfer time 120 ms. Data dependant acquisition was performed with: MS spectra rate: 2 Hz, MS/MS spectra rate: 1 Hz at 2,500 cts to 10 Hz at 250,000 cts and cycle time: 3 s. The collision energy and isolation width settings were automatically calculated using the AutoMSMS fragmentation table, absolute threshold 200 counts, preferred charge states: 2–4, singly charged ions excluded. A single MS/MS spectrum was acquired for each precursor and former target ions were excluded for 0.8 min unless the precursor intensity increased fourfold. Selected reaction monitoring cycled between the expected 2^+^ (*m/z* 631.305) and 3^+^ (*m/z* 421.2060) *m/z* values of the diagnostic peptide DEEEIEVRSR and a full scan mass spectrum in a 1 s period. The quadrupole isolation width was set at 3, and 2 *m/z* units for the 2^+^ and 3^+^ ions respectively. Collision energy was 33 for the 2*^+^* ion and 17 for the 3*^+^* ion.

Database searching Tandem mass spectral data were submitted to database searching against the human subset of the Uniprot database, appended with the sequences of the novel peptides, using a locally-running copy of the Mascot program (Matrix Science Ltd., version 2.4), through the Bruker ProteinScape interface (version 2.1). Search criteria specified: Enzyme, Trypsin or Asp-N; Fixed modifications, Carbamidomethyl (C); Variable modifications, Oxidation (M) and Deamidated (NQ); Peptide tolerance, 100 ppm for MALDI-MS and 10 ppm for LC-MS; MS/MS tolerance, 0.5 Da for MALDI-MS/MS and 0.1 Da for LC-MS/MS; Instrument, MALDI-TOF-TOF or ESI-QUAD-TOF. Results were filtered to accept only peptides with an expect score of 0.05 or better.

**Supplemental Figure legends**

**Fig.S1** A) Western blots showing three parallel gels of the same 10 plasma samples from lung cancer patients and two from individuals without disease (from Set B), probed with CIZ1b antibodies 2B and 043, and for CIZ1 exon 17. B) A different set of 5 plasma samples from lung cancer patients and 5 from individuals without disease (including one representative false positive sample, lane 4), probed with 043, and also for plasminogen (Ab98262 methods) and fibrinogen (F8512 methods).

**Fig.S2** Stability of CIZ1b in plasma and whole blood after various treatments, detected in western blot with anti-CIZ1b antibody 2B after reducing SDS-PAGE. Treatments were A) plasma after the indicated hours at 37^o^C, B) plasma after one hour at the indicated temperatures, C) plasma after freeze thaw cycles (-80^o^C for 5 minutes, followed by 20^o^C for 5 minutes) in addition to the single freeze cycle received by all samples after isolation. Over 10 cycles *p*=0.41 for CIZ1b band, and 0.49 after normalisation to the 55 kDa band, indicating little degeneration. D) Whole blood samples in lithium heparin were left unchilled (approximately 21^o^C) for the indicated times, prior to isolation of plasma and storage at -80^o^C. Comparison on the right shows no significant difference between plasma isolated immediately compared to after 24 hours, showing students t-test values for the indicated number of measurements. After treatments all samples were heated to 90^o^C for 10 minutes in E-PAGE loading buffer plus 200mM β-mercaptoethanol, separated by 8% SDS-PAGE and quantified as described previously (1). Results for the 65-70 kDa band with (closed circles) and without (open circles) normalization to the 55 kDa band are shown. Graphs show mean data (solid lines) from the indicated number of individual plasma samples (dotted lines) which were each analysed in triplicate, with SEM. Data is plotted relative to an untreated control sample in each case (once frozen plasma). Plasmas used in this series of experiments are detailed in SI Table 2.

**Fig.S3** Effect of denaturation on CIZ1b epitope. A) Left, Coomassie blue stained gel of a representative lung cancer (C) and normal (N) plasma sample (2ul), separated under fully native conditions (in the absence of SDS or reducing agent). m indicates marker lanes. Middle, parallel gel after transfer to nitrocellulose stained with Ponceau S. Right, the same membrane probed with CIZ1b antibody 043. Lower panel shows the same two samples (red arrows) separated by denaturing SDS-PAGE, also probed with 043. B) Native gel first dimension was soaked in 4x SDS-PAGE loading buffer for 30 minutes without heating, and further separated by size (second dimension). Western blot reveals one band at 55kDa that is reactive with antibody 2B in the non-cancer sample, and two bands (55 and 70 kDa) in the cancer sample. Two additional cancer plasma samples, treated in the same way are shown below. C) Migration of 043-reactive bands in a representative cancer (C) and non-cancer (N) plasma sample through a non-denaturing gel, after prior incubation at the indicated temperatures, in the presence of 2% SDS, with and without reducing agent (200mM β-mercaptoethanol, βME) as indicated. Note the shift of cancer-specific band (red box) from relative mobility of approximately 340 kDa in the absence of reducing agent, to ~70 kDa in the presence of reducing agent (accompanied by complete loss of the ‘generic’ band that was detected in all samples in the absence of reducing agent). D) Plasma from a lung cancer patient showing 043-reactive band after incubation with 1% SDS at 37^O^C for 30 mins, with the indicated concentrations of DTT, or a 10-fold excess of βME. The reactive band is indicated as it shifts from 340 kDa to 70 kDa, and is then lost under maximally reducing conditions. The behaviour of fibrinogen in the same samples is shown for comparison, detected with antibody F8512. E) Synthetic CIZ1b epitope generated by combining CIZ1b peptide (b66, SI Table 1) with normal (non-cancer) plasma (see Fig. 3), behaves similarly to endogenous epitope, dissociating from within a high molecular weight complex, to a ~70kDa species, and then disappearing under maximally reducing conditions. Fibrinogen is detected with antibody AF4786. βME concentrations refer to multiples of the standard concentration used in SDS-PAGE of 200 mM.

**Fig.S4** A) Western blots of reducing SDS-PAGE gel showing CIZ1b and the exon 17-containing species in lung cancer patient plasma, after fractionation using a total exosome isolation kit (Invitrogen 4484450 methods). Lanes show equivalent of 0.5 μl of plasma before (lane 1) and after (lane 2) addition of exosome precipitation reagent, before centrifugation (Total, T). The supernatent (SN) and pellet fraction (P) after centrifugation are in lanes 3 and 4. Lanes 5 and 6 show 1 and 2 μl equivalents of the pellet fraction respectively. Note that the CIZ1b containing species partitions with the pellet fraction while the exon 17-containing species partitions primarily with the supernatant (red arrows). B) Exosome fractionation in the presence of detergent (1% SDS), and either 2 mM EDTA or protease inhibitor cocktail (Pi) as indicated. Note the detergent-dependent shift of CIZ1b into the supernatant fraction. C) Reducing SDS-PAGE gels stained with Coomassie blue to reveal mobility of purified synthetic CIZ1 peptides. Long CIZ1b peptide (1) and CIZ1a equivalent (2) migrate with apparent molecular weight approximately equivalent to three times their formula weight, at ~21 kDa (right panel), indicating some retention of stable structure under reducing conditions. Similarly, short CIZ1b peptide (lane 3) migrates as a trimer, while equivalent sequences bearing single carboxylated glutamic acid residues at the indicated positions (4, 5, 6) migrate as expected for monomeric peptide, at approximately 1.6 kDa (left panel).

**Fig.S5** A) Sequence of CIZ1 long CIZ1b peptide (b66 - bold) plus five amino-acids of upstream sequence, showing cleavage sites for Asp-N (red) and trypsin (blue). The Asp-N peptide that spans the CIZ1b junction is indicated in red and labeled ‘diagnostic peptide’. B) MALDI-MS spectrum of Asp-N digested CIZ1b66 peptide showing peptides confirmed by MS^2^ fragmentation. Inset, MALDI-MS/MS spectrum of *m/z* 1261.6 ion identified as DEEEIEVRSR. C) Selective reaction monitoring post Asp-N digest of band P (orange, see below), and diagnostic peptide standard DEEEIEVRSR (purple) monitoring the position of the expected 2^+^ and 3^+^ ions of DEEEIEVRSR; *m/z* 631.3 and 421.2060 respectively. The top two panes show the extracted ion chromatograms (EICs) of the *m/z* 631.3. The lower two panes display the MS^1^ spectrum centered around the *m/z* of the 2^+^ ion at the apex of the EIC in the standard (36.6 mins). The expected m/z of the peptide 631.3 is clearly visible in the standard (purple) but not present above the noise in the sample derived from band P (orange). D) Denaturing SDS-PAGE of the indicated plasma/peptide mixtures, stained with Coomassie blue, before and after isolation of the indicated band ‘P’ (orange box), containing the reconstituted CIZ1b epitope. Below, CIZ1b western blot of the same samples, showing composite epitope generated by addition of long CIZ1b peptide b66 to normal plasma (lane 2), and endogenous CIZ1b band in lung cancer plasma (lane 4). Pure peptide (lane 1) migrates with three times the expected molecular mass (~21 kDa, Supplemental Fig. 4C) and is not detected by the CIZ1b antibody until complexed with carrier protein (lane 2). Note, that epitope reconstituted from peptide b66 migrates at 82 kDa, which is larger than either endogenous epitope or that created by short CIZ1b peptide b13 (see Fig. 3). Right, Ponceau S stained membrane.

**Fig.S6** ELISA optimization experiments. A) Optimal plasma concentration for discrimination of cancer and non-cancer samples via CIZ1b/fibrinogen alpha complex ELISA, is achieved with 5ul of plasma (YH 55 and 68) and unless stated otherwise is used throughout. B) Dot blot comparison showing the degree of similarity between western blot and ELISA data for development set B (R^2^ = 0.3575). After transposition of ELISA data to the same scale as western blot data by scaling to the same normal and cancer calibrator samples, paired t-test returned p= 0.501. C) Optimization of plasma concentration for quantitative detection of fibrinogen, showing ELISA output generated by the indicated concentrations of plasma. Methodology was as described except that SDS was not added to analyte buffer, and the CIZ1b capture antibody was substituted by 1ug of chick anti-fibrinogen antibody (filled circles), or no capture antibody (open circles). Detector antibody was sheep anti-fibrinogen AF4786 used as described in methods. Fibrinogen signal shows that output in the linear range is observed between 0.05 and 0.5 nanolitres of plasma. D) Effect of the CIZ1b component on ELISA reproducibility and selectivity for cancer plasma. Graph shows mean signal from two biological and two technical replicate analyses of plasma mixtures of the indicated composition, after subtraction of no plasma control readings, with SEM. Fibrinogen measurements are carried out on the same sample mixtures as CIZ1b/fibrinogen alpha complex, after dilution to accommodate the high level of fibrinogen in human plasma. Fibrinogen is detected with high sensitivity but poor reproducibility, and little quantifiable difference between lung cancer and non-cancer plasmas, while CIZ1b signal reflects the contribution of the cancer plasma to the analyte mixture. E) Limit of detection of CIZ1b by 043 measured using the indicated concentration range of purified fibrinogen with and without complexing with peptide b13 (Table S1), as indicated. CIZ1b signal is first evident above control in the presence of approximately 1 pmol of peptide epitope, and yields a quantitative response up to and beyond 300 pmols. Grey mask highlights region expanded to right.

**Table S1 Synthetic peptides**

| **Designation** | **Sequence** | **Comments** |
| --- | --- | --- |
| **Short CIZ1b (b13)** | CDEEEIE**V**RSRDIS-NH_2_ | **Observed mobility trimer** |
| **Super short CIZ1b (b7)** | CEIE**V**RSR-NH_2_ |  |
| **Short CIZ1a (a22)** | CDEEEIEVEEELCKQVRSRDISR-NH2 |  |
| **Long CIZ1b peptide (b66)** | EIAGQDEDHFITVDAVGCFEGDEEEEEDDEDEEEIE**V**RSRDISREEWKGSETYSPNTAYGVDFLVP | Observed mobility in SDS-PAGE ~25kDa |
| **Long CIZ1a peptide (a74)** | EIAGQDEDHFITVDAVGCFEGDEEEEEDDEDEEEIEVEEELCKQVRSRDISREEWKGSETYSPNTAYGVDFLVP | Observed mobility in SDS-PAGE ~25kDa |
| GLA3 | CDEEγIE**V**RSRDIS-NH_2_ | **Observed mobility monomer** |
| GLA4 | CDEγEIE**V**RSRDIS-NH_2_ | **Observed mobility monomer** |
| GLA6 | CDγγγIγ**V**RSRDIS-NH_2_ | **Observed mobility monomer** |
| Exon 17 | C-TSSGRPPSQPNTQDKTPSK  C-TARPSQPPLPRRSTRLKT | **Immunogens** |

**The 8 amino-acids spliced out of CIZ1b are underlined in non-alternatively spliced sequences, and the junction created by their absence is indicated in CIZ1b sequences by a V. Where indicated peptides contain an additional N-terminal cysteine residue, and amidation of the C-terminus. Carboxylated glutamic acid residues are indicated by γ.**

**Table S2** Samples used for stability analysis, plasma/serum comparisons, and normal and cancer calibrator pools.

| Source | Full Ref. | Internal Ref. | Age | Diagnosis | Stage |
| --- | --- | --- | --- | --- | --- |
| **Stability** |  |  |  |  |  |
| York Hospital | YH-003 | YH3 | 84 | Lung carcinoma | T1 N0 M0 |
| York Hospital | YH-004 | YH4 | 67 | Lung cancer | T4 N2 M0 |
| York Hospital | YH-007 | YH7 | 74 | Adenocarcinoma | T2 N1 M1 |
| York Hospital | YH-010 | YH10 | 70 | Lung cancer | T1 N0 M0 |
| York Hospital | YH-014 | YH14 | 64 | Respiratory failure | - |
| York Hospital | YH-015 | YH15 | 81 | Adenocarcinoma | T4 N1 M1a |
| York Hospital | YH-016 | YH16 | 68 | SCC | T3 N2 M1b |
| York Hospital | YH-017 | YH17 | 73 | Primary breast cancer | ND |
| York Hospital | YH-018 | YH18 | 54 | Lung cancer | T1 N2 M0 |
| York Hospital | YH-019 | YH19 | 90 | Lung cancer | T1 N0 M0 |
| York Hospital | YH-020 | YH20 | 65 | COPD | - |
| York Hospital | YH-035 | YH35 | 88 | Large cell Lung | T2b N0 M0 |
| York Hospital | YH-038 | YH38 | 71 | NSCLC | T3 N0/2 M0 |
| York Hospital | YH-039 | YH39 | 66 | COPD | - |
| Conversant | A1F5C85CEBE020909G6 | C25 | 74 | Adenocarcinoma | 1 |
| Conversant | A1FDC2AB005030609G6 | C33 | 57 | Adenocarcinoma | 3B |
| Conversant | A1EEF202773011909G4 | C46 | 58 | SCC | 3 |
| Conversant | A1100000318063009G6 | C49 | 58 | SCC | 3A |
| **Plasma Serum** |  |  |  |  |  |
| Bioserve | 114854-214603-6 | B6 (plasma) | 74 | Adenocarcinoma | 3A |
| Bioserve | 114854-214603-7 | B7 (Serum) | 74 | Adenocarcinoma | 3A |
| Bioserve | 118770-243524-8 | B8 (plasma) | 53 | Carcinoma | 3A |
| Bioserve | 118770-243524-9 | B9 (Serum) | 53 | Carcinoma | 3A |
| Proteogenix | 041484P | P4( Plasma) | 71 | SCC | 3A |
| Proteogenix | 041484S | P4 (Serum) | 71 | SCC | 3A |
| Proteogenix | 041489P | P9 (Plasma) | 61 | SCC | 3B |
| Proteogenix | 041489S | P9 (Serum) | 61 | SCC | 3B |
| **Normal pool** |  |  |  |  |  |
| Conversant | 248ADE3A0 | 11 | 79 | Anaemia | - |
| Conversant | 25248C949 | 12 | 36 | Anaemia | - |
| Conversant | 271645560 | 13 | 44 | Anaemia | - |
| Conversant | 2AF24AA5C | 14 | 38 | Anaemia | - |
| Conversant | 2AF3BE38D | 15 | 29 | Anaemia | - |
| Conversant | 32A8F4134 | 16 | 36 | Anaemia | - |
| Conversant | 32FAA6B2D | 17 | 49 | Anaemia | - |
| Conversant | 34EA26D8D | 18 | 41 | Anaemia | - |
| Conversant | 34EA78274 | 19 | 39 | Anaemia | - |
| Conversant | 36F1BB8DB | 20 | 50 | Anaemia | - |
| **Cancer Pool** |  |  |  |  |  |
| Conversant | 100000162 | 34 | 75 | NSCLC Adeno | IV |
| Conversant | 100000426 | 35 | 67 | NSCLC Adeno | IV |
| Conversant | 100000525 | 36 | 75 | NSCLC Adeno | IV |
| Conversant | 100001025 | 37 | 79 | NSCLC Adeno | IV |
| Conversant | 100001033 | 38 | 69 | NSCLC Adeno | IV |
| Conversant | 110025474 | 39 | 58 | NSCLC Adeno | IV |
| Conversant | C28490551 | 40 | 71 | NSCLC Adeno | IV |
| Conversant | DC1460C05 | 41 | 69 | NSCLC Adeno | IV |
| Conversant | 0AEEDB620 | 52 | 74 | NSCLC Squamous | IV |
| Conversant | 0AEEDB620 | 53 | 74 | NSCLC Squamous | IV |
| Conversant | 100000173 | 54 | 75 | NSCLC Squamous | IV |
| Conversant | D06F85E2F | 55 | 68 | NSCLC Squamous | IV |
| Conversant | EDBAA87F8 | 56 | 64 | NSCLC Squamous | IV |

Sample sets (A and B) used to evaluate assay formats are detailed in SI Xl files 1 and 2.

**Supplemental Data sets (Xls files)**

**SI Data set 1** Summary diagnoses, and assay output for patients in Set A

**SI Data set 2** Summary diagnoses, and assay output for patients in Set B
